# Supplementary material for: Epidemiology of Plasmodium malariae and Plasmodium ovale spp. in a highly malaria-endemic country: a longitudinal cohort study in Kinshasa Province, Democratic Republic of Congo
Source: medRxiv. 2023 Apr 25:2023.04.20.23288826. Preprint. [Version 1] doi: 10.1101/2023.04.20.23288826 (PMC10543032; doi:10.1101/2023.04.20.23288826)
Supplement: Supplement 1 [file media-1.pdf]

SUPPLEMENT TO:

Epidemiology of *Plasmodium malariae* and *Plasmodium ovale* spp. in a highly malaria-endemic country: A longitudinal cohort study in Kinshasa Province, Democratic Republic of Congo

Authors: Rachel Sendor[1,*], Kristin Banek[2], Melchior Mwandagalirwa Kashamuka[3], Nono Mvuama[3], Joseph A. Bala[3], Marthe Nkalani[3], Georges Kihuma[3], Joseph Atibu[3], Kyaw L. Thwai[2], W. Matthew Svec[4], Varun Goel[5], Tommy Nseka[3], Jessica T. Lin[2,6], Jeffrey A. Bailey[7], Michael Emch[1,5], Margaret Carrel[8], Jonathan J. Juliano[1,2,6], Antoinette Tshefu[3,¶], Jonathan B. Parr[2,6,¶]

Affiliations:

[1] Department of Epidemiology, Gillings School of Global Public Health, University of North Carolina at Chapel Hill, Chapel Hill, North Carolina, United States of America

[2] Institute for Global Health and Infectious Diseases, University of North Carolina at Chapel Hill, Chapel Hill, North Carolina, United States of America

[3] Ecole de Santé Publique, Faculté de Médecine, University of Kinshasa, Kinshasa, Democratic Republic of the Congo

[4] University of North Carolina at Chapel Hill, Chapel Hill, North Carolina, United States of America

[5] Department of Geography, University of North Carolina at Chapel Hill, Chapel Hill, North Carolina, United States of America

[6] Division of Infectious Diseases, School of Medicine, University of North Carolina at Chapel Hill, Chapel Hill, North Carolina, United States of America

[7] Department of Pathology and Laboratory Medicine and Center for Computational Molecular Biology, Brown University, Providence, Rhode Island, United States of America

[8] Department of Geographical and Sustainability Sciences, University of Iowa, Iowa City, Iowa, United States of America

* Corresponding author: E-mail: rachel.sendor@unc.edu (RS)

¶ AT and JBP are joint senior authors.

Short Title: Non-falciparum malaria epidemiology in the DRC

Supplementary Table 1. PCR primers, probes, and assay conditions

	**Duplex Assay: *P.* malariae-specific, and *P. ovale*-specific, 18S rRNA**		
Adapted from:	Rougemont M et al. *J Clin Microbiology 2004*. 42(12):5636-43.		
**Forward Primer – *Pm*** (5'->3')	CCG ACT AGG TGT TGG ATG ATA GAG TAA A		
**Reverse Primer – *Pm*** (5'->3')	AAC CCA AAG ACT TTG ATT TCT CAT AA		
**Forward Primer – *Po*** (5'->3')	CCG ACT AGG TTT TGG ATG AAA GAT TTT T		
**Reverse Primer – *Po*** (5'->3')	AAC CCA AAG ACT TTG ATT TCT CAT AA		
**Probe – *Pm*** (5'->3')	FAM-CTA TCT AAA AGA AAC ACT CAT-MGBNFQ		
**Probe – *Po*** (5'->3')	VIC-CGA AAG GAA TTT TCT TAT T-MGBNFQ		
**Cycling conditions:**	Temp	Duration	No. Cycles
	50C	2 min	x1
	95C	10 min	x1
	95C	15 sec	x45
	60C	1 min	
Reaction conditions:	Roche FastStart Universal Probe Master (Rox)		
	Fwd primers	20µM	
	Rev primers	20µM	
	Probes	20µM	
	DNA	2 µL	
	Total volume	12 µL	
	***P. falciparum*-specific LDH**		
Adapted from:	Pickard AL et al. Antimicrob. Agents Chemo 2003. 47(8):2418-2423.		
Forward Primer (5'->3')	ACGATTTGGCTGGAGCAGAT		
Reverse Primer (5'->3')	TCTCTATTCCATTCTTTGTCACTCTTTC		
Probe (5'->3')	FAM/ AGTAATAGTAACAGCTGGATTTACCAAGGCCCCA /TAMRA		
Cycling conditions:	Temp	Duration	No. Cycles
	50C	2 min	x1
	95C	10 min	x1
	95C	15 sec	x40
	60C	1 min	
Reaction conditions:	Roche FastStart Universal Probe Master (Rox)		
	Fwd primer	200nM	
	Rev primer	200nM	
	Probe	100nM	
	Template	2 µl	
	Total volume	12 µl	

[1.]Duplex PCR was carried out to 45 cycle thresholds for *P. malariae* and *P. ovale* spp. detection; however, samples were considered positive if amplification under 40 Cts only, due to observed variability in this assay at later cycle thresholds.

Supplementary Table 2. Comparison of participant characteristics between survey population and clinic subpopulation.

Participant Baseline Characteristics *no. (%)*	Population Type[1]				
	Survey-based Population	Also in Clinic-based Subpopulation?			
	n=1,565 participants	Yes n=1,050 (67.1%)	No n=515 (32.9%)	p-value[3]	SMD
Age at visit (years)					
<5	302 (19.3)	229 (21.8)	73 (14.2)	<0.001	0.276
5-14	500 (31.9)	354 (33.7)	146 (28.3)		
15+	763 (48.8)	467 (44.5)	296 (57.5)		
Missing	0	0	0		
Sex					
Female	863 (55.1)	587 (55.9)	276 (53.6)	0.388	0.046
Male	702 (44.9)	463 (44.1)	239 (46.4)		
Missing	0	0	0		
Site					
Voix de Peuple	385 (24.6)	230 (21.9)	155 (30.1)	<0.001	0.351
Bu	209 (13.4)	171 (16.3)	38 (7.4)		
Impuru	211 (13.5)	136 (13.0)	75 (14.6)		
Pema	243 (15.5)	180 (17.1)	63 (12.2)		
Kimpoko	167 (10.7)	111 (10.6)	56 (10.9)		
Ngamanzo	258 (16.5)	167 (15.9)	91 (17.7)		
Iye	92 (5.9)	55 (5.2)	37 (7.2)		
Missing	0	0	0		
Rurality (by health area)					
Urban	385 (24.6)	230 (21.9)	155 (30.1)	<0.001	0.262
Peri-urban	517 (33.0)	333 (31.7)	184 (35.7)		
Rural	663 (42.4)	487 (46.4)	176 (34.2)		
Missing	0	0	0		
Wealth quintile					
Poorest	318 (20.3)	226 (21.5)	92 (17.9)	0.011	0.194
Poorer	314 (20.1)	209 (19.9)	105 (20.4)		
Average	311 (19.9)	223 (21.2)	88 (17.1)		
Wealthier	304 (19.4)	202 (19.2)	102 (19.8)		
Wealthiest	318 (20.3)	190 (18.1)	128 (24.9)		
Missing	0	0	0		
Fever at Baseline[2]					
Yes	382 (24.5)	272 (26.0)	110 (21.5)	0.052	0.106
No	1176 (75.5)	774 (74.0)	402 (78.5)		
Missing	7	4	3		
RDT+ at Baseline					
Yes	429 (27.4)	285 (27.2)	144 (28.0)	0.77	0.019
No	1134 (72.6)	764 (72.8)	370 (72.0)		
Missing	2	1	1		
Seasonality of visit					
Rainy	1180 (75.4)	820 (78.1)	360 (69.9)	0.001	0.188
Dry	385 (24.6)	230 (21.9)	155 (30.1)		

Participant Baseline Characteristics *no. (%)*	Survey-based Population n=1,565 participants	Also in Clinic-based Subpopulation? Yes n=1,050 (67.1%)	No n=515 (32.9%)	p-value[3]	SMD
Missing	0	0	0		
Slept under bednet at Baseline					
Yes	705 (45.0)	480 (45.7)	225 (43.7)	0.482	0.041
No	860 (55.0)	570 (54.3)	290 (56.3)		
Missing	0	0	0		
Antimalarials taken within prior 6 months					
Yes	394 (25.4)	277 (26.6)	117 (22.8)	0.119	0.088
No	1160 (74.6)	764 (73.4)	396 (77.2)		
Missing	11	9	2		
P. falciparum PCR +					
Yes	484 (30.9)	314 (29.9)	170 (33.0)	0.222	0.067
Mixed-species	*35 (7.2)*	*25 (8.0)*	*10 (5.9)*	*0.51*	*0.082*
Single-species	*449 (92.8)*	*289 (92.0)*	*160 (94.1)*		
No	1081 (69.1)	736 (70.1)	345 (67.0)		
Missing	0	0	0		
P. malariae PCR +					
Yes	47 (3.0)	33 (3.1)	14 (2.7)	0.753	0.025
Mixed-species	*31 (66.0)*	*22 (66.7)*	*9 (64.3)*	*1*	*0.05*
Single-species	*16 (34.0)*	*11 (33.3)*	*5 (35.7)*		
No	1518 (97.0)	1017 (96.9)	501 (97.3)		
Missing	0	0	0		
P. ovale spp. PCR +					
Yes	6 (0.4)	5 (0.5)	1 (0.2)	0.67	0.049
Mixed-species	*4 (66.7)*	*3 (60.0)*	*1 (100.0)*	*1*	*1.155*
Single-species	*2 (33.3)*	*2 (40.0)*	*0 (0.0)*		
No	1559 (99.6)	1045 (99.5)	514 (99.8)		
Missing	0	0	0		

Population Type[1]

[1] The survey-based population comprises all participants in the study, as all participants completed the Baseline household survey. The clinic-based sub-population comprises a subset of the survey-based population who had at least 1 symptomatic clinic visit during the study period.
[2] Fever at Baseline survey was self-reported as "fever in the prior week"
[3] p-values compare baseline characteristics between those in the survey-based population who were vs. were not also included in the clinic-based symptomatic population.

Supplemental Table 3. Baseline subject characteristics by species for the symptomatic clinic subpopulation

Baseline Participant Characteristics	Clinic-based Pop.	Baseline Malaria Infection by Species					
		P. malariae		*P. ovale* spp.		*P. falciparum*	
		PCR Pos.	PCR Neg.	PCR Pos.	PCR Neg.	PCR Pos.	PCR Neg.
no. (%)	N=1,050	n=33	n=1,017	n=5	n=1,045	n=314	n=736
**Age (years)**							
<5	229 (21.8)	4 (12.1)	225 (22.1)	1 (20.0)	228 (21.8)	49 (15.6)	180 (24.5)
5-14	354 (33.7)	23 (69.7)	331 (32.5)	2 (40.0)	352 (33.7)	161 (51.3)	193 (26.2)
15+	467 (44.5)	6 (18.2)	461 (45.3)	2 (40.0)	465 (44.5)	104 (33.1)	363 (49.3)
**Sex**							
Female	587 (55.9)	19 (57.6)	568 (55.9)	5 (100.0)	582 (55.7)	165 (52.5)	422 (57.3)
Male	463 (44.1)	14 (42.4)	449 (44.1)	0 (0.0)	463 (44.3)	149 (47.5)	314 (42.7)
**Urbanicity**							
Rural	487 (46.4)	21 (63.6)	466 (45.8)	5 (100.0)	482 (46.1)	197 (62.7)	290 (39.4)
Peri-urban	333 (31.7)	10 (30.3)	323 (31.8)	0 (0.0)	333 (31.9)	110 (35.0)	223 (30.3)
Urban	230 (21.9)	2 (6.1)	228 (22.4)	0 (0.0)	230 (22.0)	7 (2.2)	223 (30.3)
**Fever (≤1 week)**							
Yes	272 (26.0)	9 (28.1)	263 (25.9)	4 (80.0)	268 (25.7)	110 (35.3)	162 (22.1)
**RDT+**							
Yes	285 (27.2)	20 (62.5)	265 (26.1)	2 (40.0)	283 (27.1)	235 (75.1)	50 (6.8)
**Bed Net Use (Prior Night)**							
Yes	480 (45.7)	9 (27.3)	471 (46.3)	2 (40.0)	478 (45.7)	127 (40.4)	353 (48.0)
**Symptoms in Prior 6 mon.**							
Yes	269 (25.7)	7 (21.9)	262 (25.8)	3 (60.0)	266 (25.5)	81 (26.0)	188 (25.6)
**Tx with Antimalarials in Prior 6 mon.**							
Yes	277 (26.6)	7 (21.9)	270 (26.8)	1 (20.0)	276 (26.6)	76 (24.4)	201 (27.6)
**Wealth Category**							
Poorest	226 (21.5)	5 (15.2)	221 (21.7)	1 (20.0)	225 (21.5)	93 (29.6)	133 (18.1)
Poorer	209 (19.9)	14 (42.4)	195 (19.2)	0 (0.0)	209 (20.0)	78 (24.8)	131 (17.8)
Average	223 (21.2)	6 (18.2)	217 (21.3)	2 (40.0)	221 (21.1)	69 (22.0)	154 (20.9)
Wealthier	202 (19.2)	6 (18.2)	196 (19.3)	2 (40.0)	200 (19.1)	69 (22.0)	133 (18.1)
Wealthiest	190 (18.1)	2 (6.1)	188 (18.5)	0 (0.0)	190 (18.2)	5 (1.6)	185 (25.1)

Supplemental Table 4. Participant characteristics across follow-up visits (Survey population)

Participant characteristics at each visit *no. (%)*	Household Survey Visits (*Active surveillance*) Baseline n=1,565	Follow-up 1 n=1,447	Follow-up 2 n=1,367	Follow-up 3 n=1,303	p-value[3]
No. subjects	1,565	1,447	1,367	1,303	
Age at visit (years)					
<5	302 (19.3)	271 (18.7)	225 (16.5)	185 (14.2)	0.009
5-14	500 (31.9)	480 (33.2)	443 (32.4)	453 (34.8)	
15+	763 (48.8)	696 (48.1)	699 (51.1)	665 (51.0)	
Missing	*0*	*0*	*0*	*0*	
Sex					
Female	863 (55.1)	800 (55.3)	757 (55.4)	707 (54.3)	0.935
Male	702 (44.9)	647 (44.7)	610 (44.6)	596 (45.7)	
Missing	*0*	*0*	*0*	*0*	
Rurality (by health area)					
Urban	385 (24.6)	342 (23.6)	307 (22.5)	300 (23.0)	0.384
Peri-urban	517 (33.0)	487 (33.7)	457 (33.4)	402 (30.9)	
Rural	663 (42.4)	618 (42.7)	603 (44.1)	601 (46.1)	
Missing	*0*	*0*	*0*	*0*	
Wealth quintile					
Poorest	318 (20.3)	294 (20.3)	290 (21.2)	274 (21.0)	0.994
Poorer	314 (20.1)	288 (19.9)	277 (20.3)	264 (20.3)	
Average	311 (19.9)	301 (20.8)	292 (21.4)	271 (20.8)	
Wealthier	304 (19.4)	284 (19.6)	255 (18.7)	249 (19.1)	
Wealthiest	318 (20.3)	280 (19.4)	253 (18.5)	245 (18.8)	
Missing	*0*	*0*	*0*	*0*	
Fever[1]					
Yes	382 (24.5)	173 (12.0)	249 (18.2)	120 (9.2)	<0.001
No	1176 (75.5)	1273 (88.0)	1118 (81.8)	1183 (90.8)	
Missing	*7*	*1*	*0*	*0*	
RDT+					
Yes	429 (27.4)	396 (27.4)	511 (37.4)	307 (23.6)	<0.001
No	1134 (72.6)	1051 (72.6)	856 (62.6)	995 (76.4)	
Missing	*2*	*0*	*0*	*1*	
Slept under bednet the prior night					
Yes	705 (45.0)	810 (56.0)	732 (53.5)	589 (45.2)	<0.001
No	860 (55.0)	637 (44.0)	635 (46.5)	714 (54.8)	
Missing	*0*	*0*	*0*	*0*	
Seasonality of visit					
Rainy	1180 (75.4)	0 (0.0)	1365 (99.9)	19 (1.5)	<0.001
Dry	385 (24.6)	1447 (100.0)	2 (0.1)	1284 (98.5)	
Missing	*0*	*0*	*0*	*0*	
Antimalarials taken within prior 6 months[2]					
Yes	394 (25.4)	559 (38.9)	625 (46.1)	569 (46.6)	<0.001
No	1160 (74.6)	877 (61.1)	732 (53.9)	652 (53.4)	
Missing	*11*	*11*	*10*	*82*	
P. falciparum PCR +					
Yes	484 (30.9)	512 (35.5)	538 (39.6)	442 (34.1)	<0.001

Participant characteristics at each visit *no. (%)*	Household Survey Visits (*Active surveillance)* Baseline n=1,565	Follow-up 1 n=1,447	Follow-up 2 n=1,367	Follow-up 3 n=1,303	p-value[3]
Mixed-species	*35 (7.2)*	*42 (8.2)*	*64 (11.9)*	*47 (10.6)*	*0.043* [4]
Single-species	*449 (92.8)*	*470 (91.8)*	*474 (88.1)*	*395 (89.4)*	
No	1081 (69.1)	930 (64.5)	820 (60.4)	853 (65.9)	
Missing	*0*	*5*	*9*	*8*	
P. malariae PCR +					
Yes	47 (3.0)	35 (2.4)	56 (4.1)	48 (3.7)	0.055 [4]
Mixed-species	*31 (66.0)*	*24 (68.6)*	*44 (78.6)*	*38 (79.2)*	*0.346* [4]
Single-species	*16 (34.0)*	*11 (31.4)*	*12 (21.4)*	*10 (20.8)*	
No	1518 (97.0)	1408 (97.6)	1300 (95.9)	1247 (96.3)	
Missing	*0*	*4*	*11*	*8*	
P. ovale spp. PCR +					
Yes	6 (0.4)	27 (1.9)	27 (2.0)	18 (1.4)	<0.001 [4]
Mixed-species	*4 (66.7)*	*20 (74.1)*	*24 (88.9)*	*13 (72.2)*	*0.358* [4]
Single-species	*2 (33.3)*	*7 (25.9)*	*3 (11.1)*	*5 (27.8)*	
No	1559 (99.6)	1416 (98.1)	1329 (98.0)	1277 (98.6)	
Missing	*0*	*4*	*11*	*8*	

[1] Fever was self-reported as "fever in the prior week"

[2] Self-reported use of antimalarials in prior 6 months.

[3] Categorical variables were statistically compared using chi-squared tests; continuous variables were compared using the Kruskal-Wallis test of medians to account for non-normality. Missing data were excluded from statistical tests.

[4] p-values tested using Fisher's exact testing due to small cell sizes.

Supplemental Table 5. Participant characteristics across follow-up (Clinic Subpopulation)

Participant characteristics at visits *no. (%)*	Clinic Visits			
	Time from Baseline Visit[2]			
	**All visits within first 12 months** n=1297	**All visits between 12 to 24 months** n=1203	**All visits after 24 months** n=907	p-value[3]
No. subjects with visits	732	648	510	
Age at visit (years)				
<5	353 (27.2)	305 (25.4)	172 (19.0)	<0.001
5-14	481 (37.1)	434 (36.1)	386 (42.6)	
15+	463 (35.7)	462 (38.5)	348 (38.4)	
Missing	*0*	*2*	*1*	
Sex				
Female	741 (57.1)	726 (60.3)	514 (56.7)	0.153
Male	556 (42.9)	477 (39.7)	393 (43.3)	
Missing	*0*	*0*	*0*	
Site				
Voix de Peuple	182 (14.0)	213 (17.7)	109 (12.0)	<0.001
Bu	232 (17.9)	305 (25.4)	236 (26.0)	
Impuru	177 (13.6)	139 (11.6)	73 (8.0)	
Pema	261 (20.1)	203 (16.9)	162 (17.9)	
Kimpoko	217 (16.7)	114 (9.5)	104 (11.5)	
Ngamanzo	181 (14.0)	187 (15.5)	190 (20.9)	
Iye	47 (3.6)	42 (3.5)	33 (3.6)	
Missing	*0*	*0*	*0*	
Rurality (by health area)				
Urban	182 (14.0)	213 (17.7)	109 (12.0)	<0.001
Peri-urban	445 (34.3)	343 (28.5)	327 (36.1)	
Rural	670 (51.7)	647 (53.8)	471 (51.9)	
Missing	*0*	*0*	*0*	
Wealth quintile				
Poorest	288 (22.2)	235 (19.5)	201 (22.2)	<0.001
Poorer	267 (20.6)	309 (25.7)	230 (25.4)	
Average	332 (25.6)	279 (23.2)	232 (25.6)	
Wealthier	267 (20.6)	192 (16.0)	156 (17.2)	
Wealthiest	143 (11.0)	188 (15.6)	88 (9.7)	
Missing	*0*	*0*	*0*	
Fever[1]				
Yes	649 (75.1)	384 (48.7)	274 (44.4)	<0.001 [4]
No	215 (24.9)	404 (51.3)	343 (55.6)	
Missing	*433*	*415*	*290*	
RDT+				
Yes	1145 (88.4)	948 (79.0)	687 (76.1)	<0.001 [4]
No	150 (11.6)	252 (21.0)	216 (23.9)	
Missing	*2*	*3*	*4*	
Anemia				
Severe	48 (4.1)	32 (2.7)	17 (1.9)	0.009
Moderate	249 (21.4)	230 (19.4)	167 (18.8)	
Mild	202 (17.4)	242 (20.4)	197 (22.2)	
Not anemic	662 (57.0)	681 (57.5)	506 (57.0)	

Participant characteristics at visits *no. (%)*	Clinic Visits — Time from Baseline Visit[2]: All visits within first 12 months n=1297	All visits between 12 to 24 months n=1203	All visits after 24 months n=907	p-value[3]
Missing	*136*	*18*	*20*	
Seasonality of visit				
Rainy	885 (68.2)	758 (63.0)	411 (45.3)	<0.001 [4]
Dry	412 (31.8)	445 (37.0)	496 (54.7)	
Missing	*0*	*0*	*0*	
P. falciparum PCR +				
Yes	829 (64.6)	636 (53.5)	544 (62.0)	<0.001
Mixed-species	*53 (6.4)*	*42 (6.6)*	*41 (7.5)*	*0.694* [4]
Single-species	*775 (93.6)*	*593 (93.4)*	*503 (92.5)*	
No	455 (35.4)	552 (46.5)	334 (38.0)	
Missing	*13*	*15*	*29*	
P. malariae PCR +				
Yes	53 (4.1)	44 (3.7)	38 (4.3)	0.757 [4]
Mixed-species	*35 (66.0)*	*25 (56.8)*	*29 (76.3)*	*0.187* [4]
Single-species	*18 (34.0)*	*19 (43.2)*	*9 (23.7)*	
No	1230 (95.9)	1142 (96.3)	842 (95.7)	
Missing	*14*	*17*	*27*	
P. ovale spp. PCR +				
Yes	34 (2.7)	41 (3.5)	20 (2.3)	0.255 [4]
Mixed-species	*19 (55.9)*	*21 (51.2)*	*12 (60.0)*	*0.864* [4]
Single-species	*15 (44.1)*	*20 (48.8)*	*8 (40.0)*	
No	1249 (97.3)	1145 (96.5)	860 (97.7)	
Missing	*14*	*17*	*27*	

[1] Fever was measured at time of clinic visit.

[2] Clinic visits could continue past the end of active surveillance till the end of 2017, for a total of 34 months from the first baseline visit. Clinic visits after 24 months occurred after all household surveys had concluded.

[3] Categorical variables were statistically compared using chi-squared tests; continuous variables were compared using the Kruskal-Wallis test of medians to account for non-normality. Missing data were excluded from statistical tests.

[4] p-values tested using Fisher's exact testing due to small cell sizes.

Supplemental Figure 1 A-B.) Factors associated with *P. malariae* and *P. ovale* spp. infection prevalence, compared to *P. falciparum,* stratified by study population.

A.

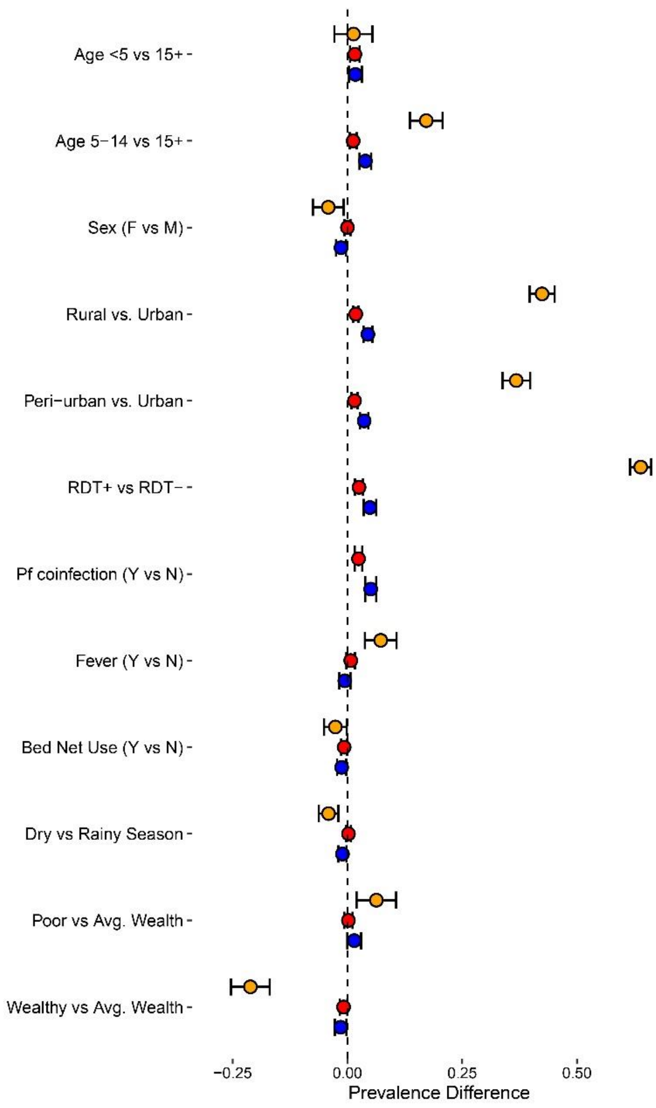

B.

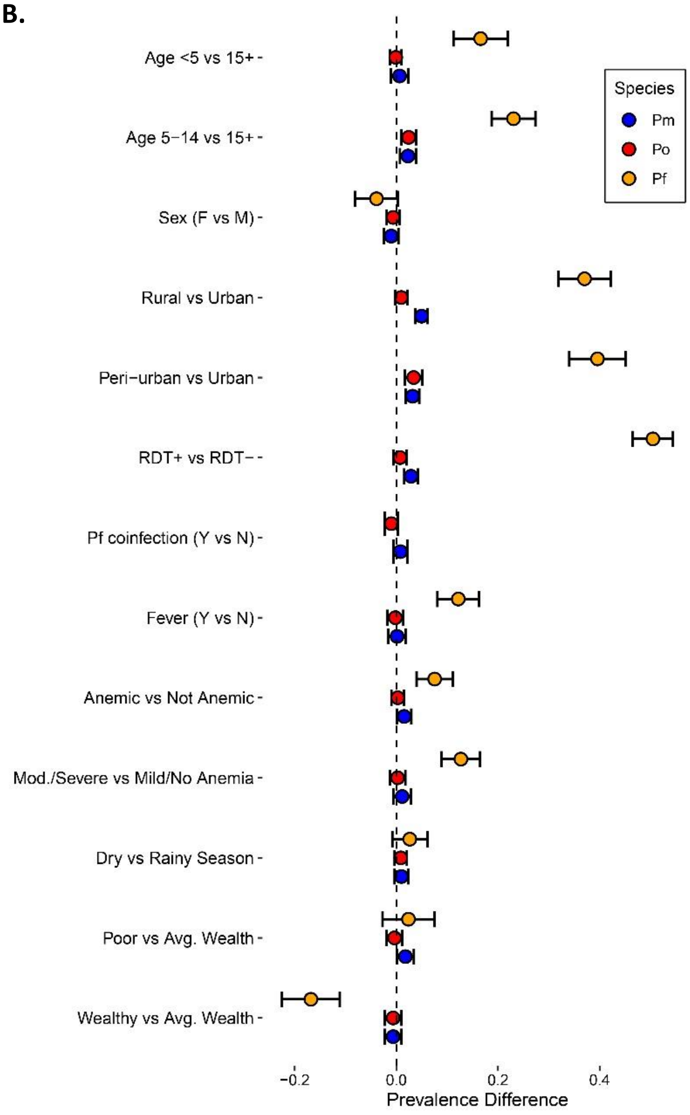

A) Factors associated with non-falciparum infection prevalence compared to *P. falciparum* infection, <u>at survey visits</u> (baseline and three follow-up surveys). B) Factors associated with non-falciparum infection prevalence compared to *P. falciparum* infection, at <u>clinic</u> visits throughout follow-up.

Supplemental Figures 2 A-D. *P. malariae* and *P. ovale* spp. infections throughout the study period, within the Total Population, encompassing infections detected at all touch points in the study (survey + clinic visits).

A.

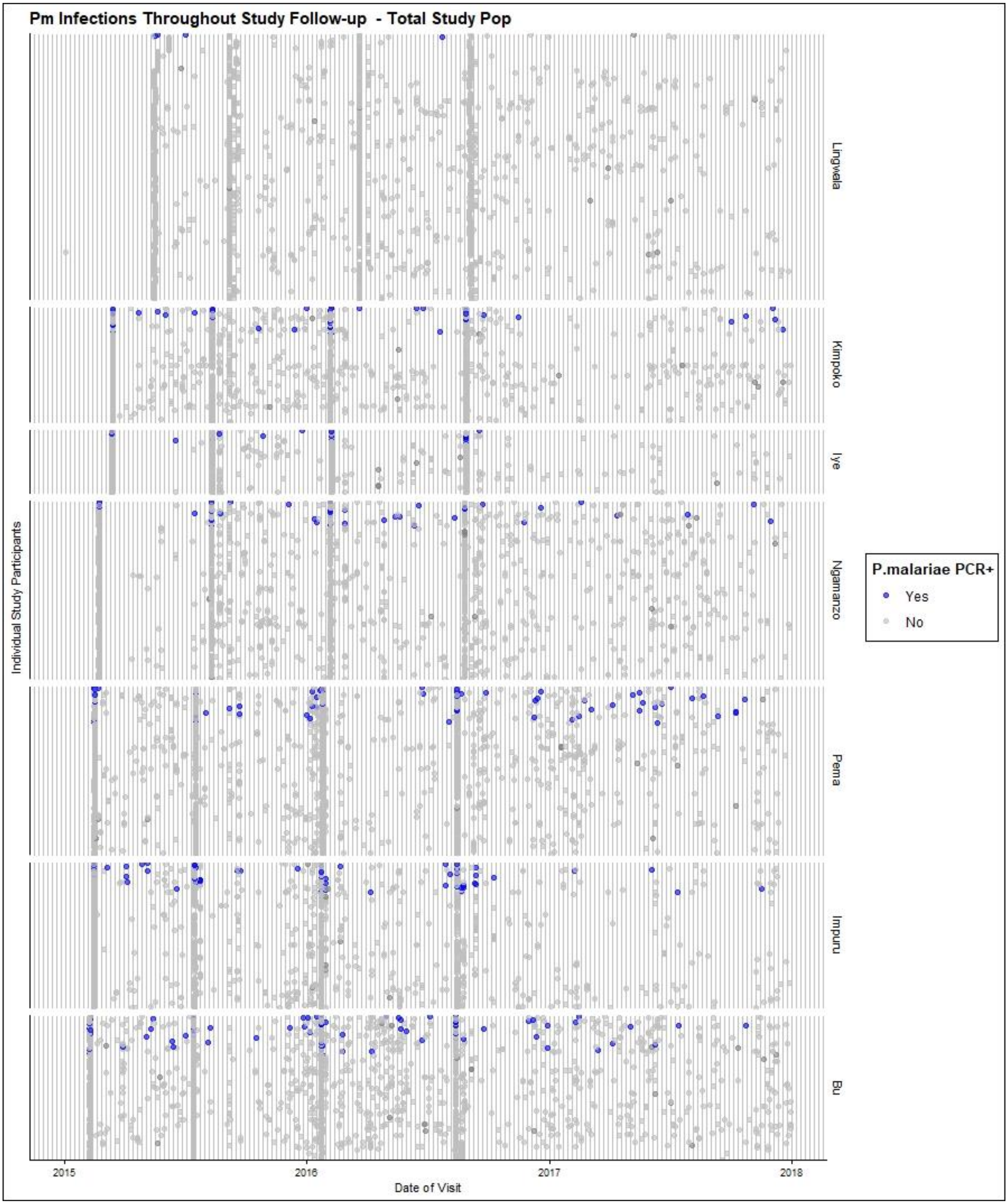

A) *P. malariae* infections detected at study visits throughout follow-up, among all subjects in the Total Population. Rows represent individual subjects, sorted by frequency of PCR+ *P. malariae* infections throughout the full study period.

B

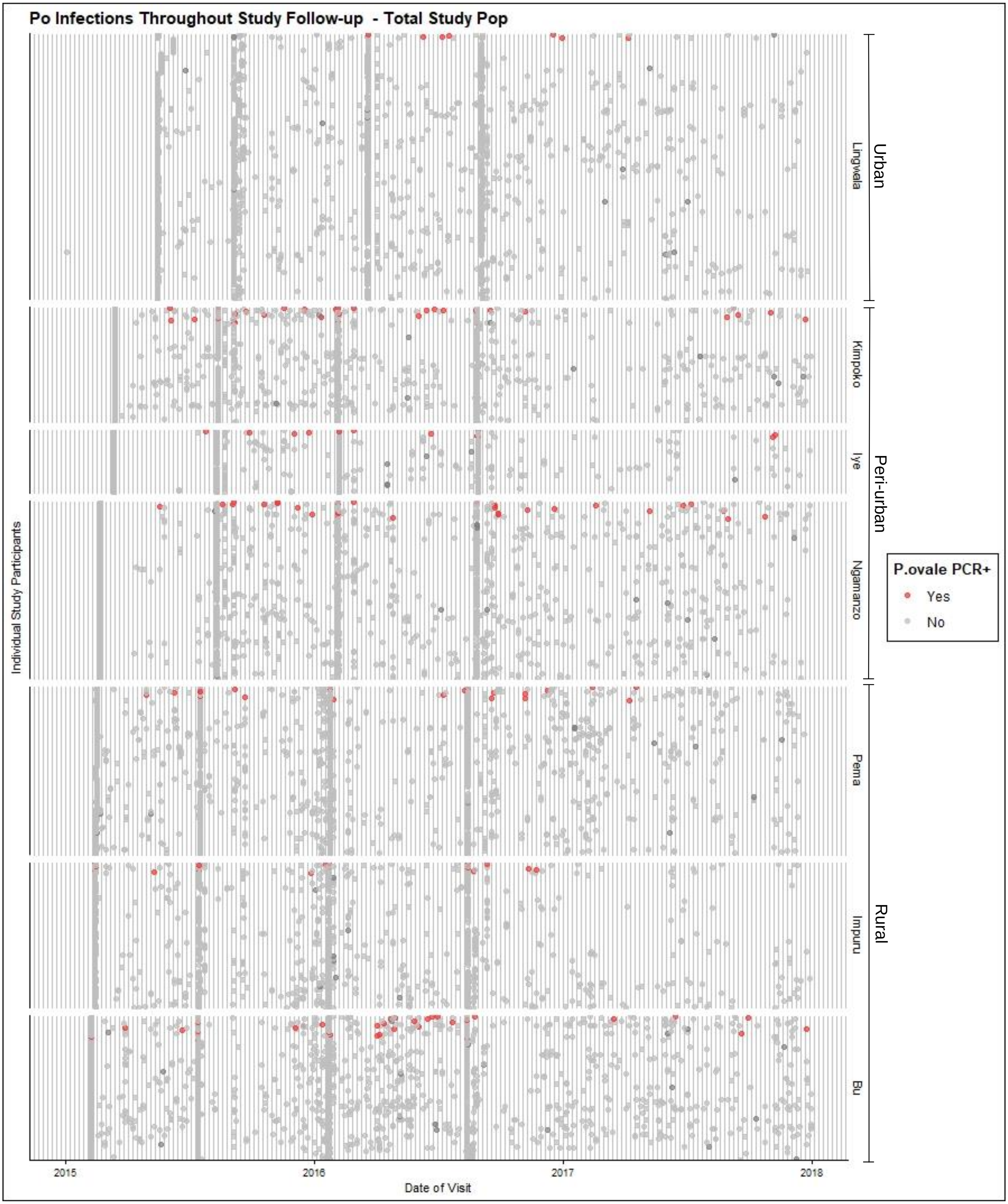

B) *P. ovale* spp. infections detected at study visits throughout follow-up, among all subjects in the Total Population. Rows represent individual subjects, sorted by frequency of PCR+ *P. ovale* spp. infections throughout the full study period.

C

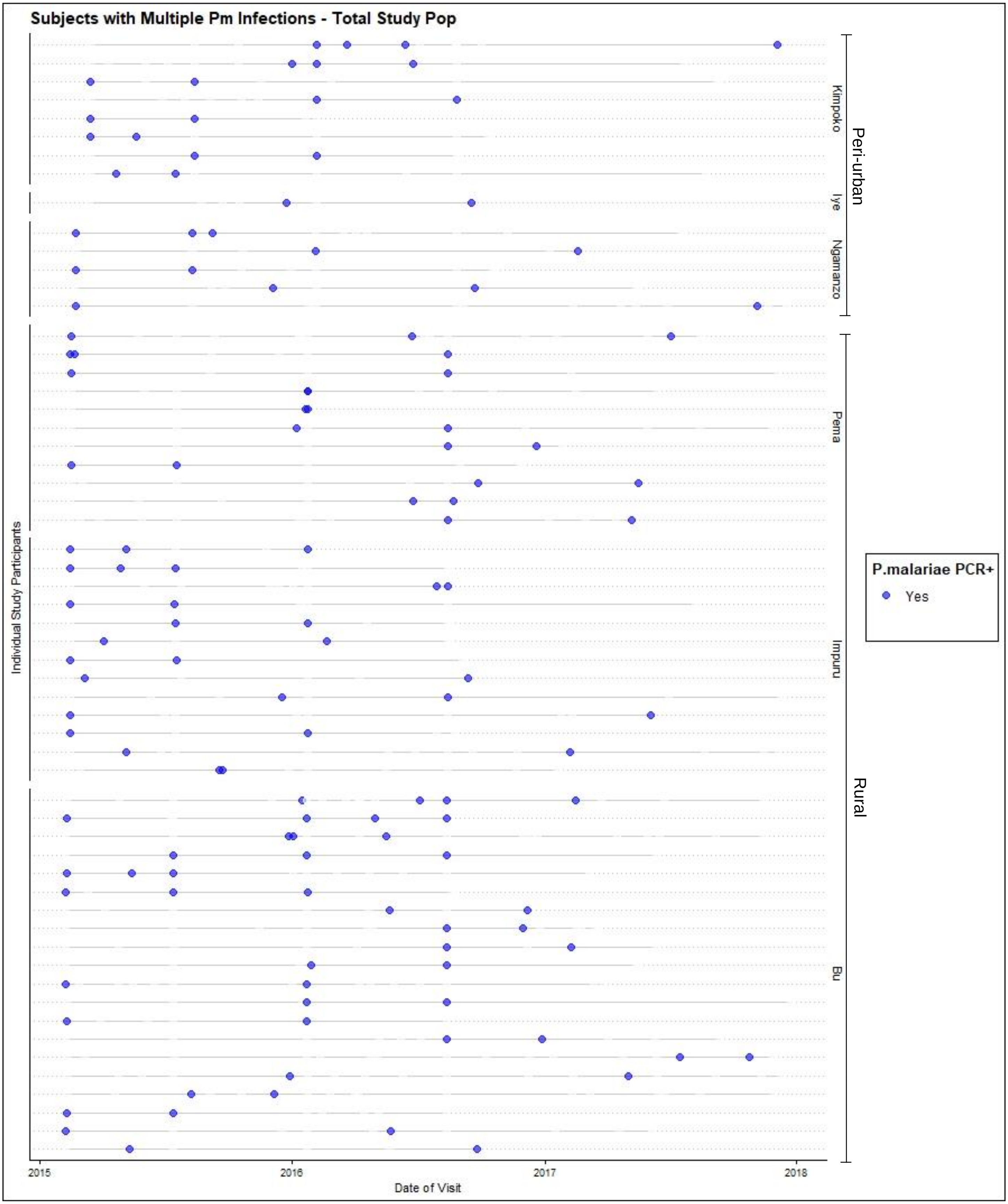

C) Multiple *P. malariae* infections detected at study visits throughout follow-up, among all subjects in the Total Population who had at least one *P.* malariae infection during the study. Rows represent individual subjects, sorted by frequency of PCR+ *P. malariae* spp. infections throughout the full study period.

D

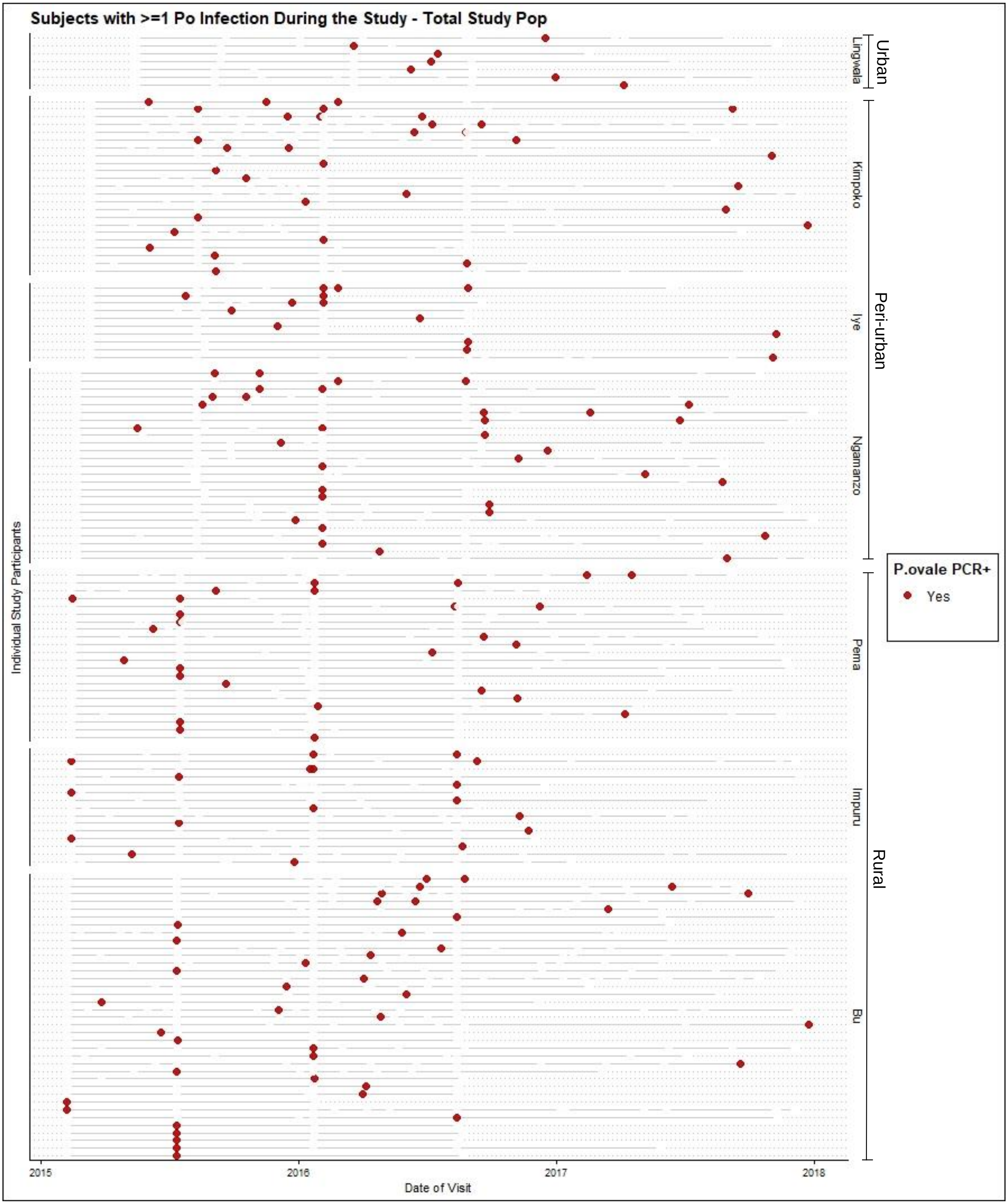

D) Multiple *P. ovale* spp. infections detected at study visits throughout follow-up, among all subjects in the Total Population <u>who had at least one *P.* ovale spp. infection during the study</u>. Rows represent individual subjects, sorted by frequency of PCR+ *P. ovale* spp. infections throughout the full study period.

Supplemental Table 6. Estimated parasitemias (p/µL) by malaria species

		**Survey Population** – *Household Survey Visits*					
**Parasitemia Estimates (p/uL)**		n (infect.)	median	IQR	min - max	p-value	n excluded (rehydrated)‡
**Pm (p/uL)**	Total	175	22.4	8.5-72.2	0.8-358,246		11
	Mixed	129	21.1	8.5-60.1	1.4-358,246	0.259	8
	Mono	46	28.8	9.2-125	0.8-688		3
**Po (p/uL)**	Total	71	5.8	2.0-28.0	0.6-106,476		7
	Mixed	56	5.7	2.3-23.0	0.6-106,476	0.662	5
	Mono	15	9.5	1.7-92.2	0.8-252		2
**Pf (p/uL)**	Total	1760	52.6	8.2-343.3	0.6-268250.0		216
	Mixed	176	120.5	31.9-496.3	0.8-22925.0	<0.001	12
	Mono	1584	46.9	7.4-311.1	0.6-268250.0		204

		**Clinic sub-Population** – *Symptomatic Clinic Visits*					
**Parasitemia Estimates (p/uL)**		n (infect.)	median	IQR	min - max	p-value	n excluded (rehydrated)
**Pm (p/uL)**	Total	132	36.5	4.7-182	0.6-23,288		3
	Mixed	88	31.8	3.6-157	0.6-2,432	0.239	1
	Mono	44	51.5	15.2-204	1.3-23,288		2
**Po (p/uL)**	Total	93	17.7	4.6-65.8	0.2-2,875		2
	Mixed	51	17.8	4.9-68.6	0.3-1,447	0.740	1
	Mono	42	16.7	4.5-65.5	0.2-2,875		1
**Pf (p/uL)**	Total	1970	2644.3	113.2-16931.1	0.6-1,165,100		39
	Mixed	134	508.7	58.8-8387.1	2.1-119,100	0.001	2
	Mono	1834	2897	126.5-18058.3	0.6-1,165,100		37

		**Total Population = Survey and Clinic Visits**					
**Parasitemia Estimates (p/uL)**		n (infect.)	median	IQR	min - max	p-value	n excluded (rehydrated)
**Pm (p/uL)**	Total	307	25.7	7.7-119	0.6-358,246		14
	Mixed	217	22.4	6.9-108	0.6-358,246	0.071	9
	Mono	90	36.5	11.8 -187	0.8-23,288		5
**Po (p/uL)**	Total	164	10.2	2.7-47.4	0.2-106,476		9
	Mixed	107	10.8	2.8-36.4	0.3-106.476	0.465	6
	Mono	57	15.8	2.2-65.8	0.2-2,875		3
**Pf (p/uL)**	Total	3730	266.5	18.8-4525.5	0.6-1,165,100		255
	Mixed	310	190	40.7-1427.4	0.8-119,100	0.209	14
	Mono	3418	279.6	17.4-5014.6	0.6-1,165,100		241
